# Context-specific comparison of sleep acquisition systems in *Drosophila*

**DOI:** 10.1242/bio.013011

**Published:** 2015-10-30

**Authors:** David S. Garbe, Wesley L. Bollinger, Abigail Vigderman, Pavel Masek, Jill Gertowski, Amita Sehgal, Alex C. Keene

**Affiliations:** 1 Department of Neuroscience, Perelman School of Medicine at the University of Pennsylvania, Philadelphia, PA 19104, USA; 2 Department of Biology, University of Nevada-Reno, Reno, NV 89557, USA; 3 Department of Biological Sciences, Florida Atlantic University, 5353 Parkside Drive, Jupiter, FL 33458, USA; 4 Howard Hughes Medical Institute, Department of Neuroscience, Perelman School of Medicine at the University of Pennsylvania, Philadelphia, PA 19104, USA; 5 Department of Biology, SUNY Binghamton, Binghamton, NY 13902, USA

**Keywords:** *Drosophila*, Sleep, Video tracking, Place preference

## Abstract

Sleep is conserved across phyla and can be measured through electrophysiological or behavioral characteristics. The fruit fly, *Drosophila melanogaster*, provides an excellent model for investigating the genetic and neural mechanisms that regulate sleep. Multiple systems exist for measuring fly activity, including video analysis and single-beam (SB) or multi-beam (MB) infrared (IR)-based monitoring. In this study, we compare multiple sleep parameters of individual flies using a custom-built video-based acquisition system, and commercially available SB- or MB-IR acquisition systems. We report that all three monitoring systems appear sufficiently sensitive to detect changes in sleep duration associated with diet, age, and mating status. Our data also demonstrate that MB-IR detection appeared more sensitive than the SB-IR for detecting baseline nuances in sleep architecture, while architectural changes associated with varying life-history and environment were generally detected across all acquisition types. Finally, video recording of flies in an arena allowed us to measure the effect of ambient environment on sleep. These experiments demonstrate a robust effect of arena shape and size as well as light levels on sleep duration and architecture, and highlighting the versatility of tracking-based sleep acquisition. These findings provide insight into the context-specific basis for choosing between *Drosophila* sleep acquisition systems, describe a novel cost-effective system for video tracking, and characterize sleep analysis using the MB-IR sleep analysis. Further, we describe a modified dark-place preference sleep assay using video tracking, confirming that flies prefer to sleep in dark locations.

## Abstract (precis)

**Summary:** Multiple sleep acquisition systems provide efficient and accurate methods for measuring sleep duration in *Drosophila*, while video tracking and multibeam analysis allow for increased flexibility of behavioral paradigms and greater resolution of sleep architecture.

## INTRODUCTION

A number of behavioral criteria have been used to define sleep in model organisms, including consolidated periods of immobility, increased arousal threshold, a species-specific stereotyped posture, decreased brain activity, and rebound after sleep deprivation (Hendricks et al., 2000; Zimmerman et al., 2008; Shaw et al., 2000; Zhdanova et al., 2001; Raizen et al., 2008). These models are proving to be extremely useful as their genetic amenability is leading to the identification of genes and molecules involved in sleep-wake regulation (Sehgal and Mignot, 2011; Allada and Siegel, 2008; Cirelli, 2009; Griffith, 2013). *Drosophila*, in particular, is an attractive model that displays electrophysiological and behavioral changes typical of sleep. Importantly, many genetic and neural principles underlying sleep-wake regulation are conserved between *Drosophila* and mammals (Griffith, 2013; Sehgal and Mignot, 2011).

In *Drosophila*, changes in arousal threshold and brain activity are observed during immobility bouts lasting ≥5 min; therefore, this threshold has been the most commonly used parameter for quantifying sleep (Shaw et al., 2000; Van Alphen et al., 2013; Faville et al., 2015). Traditionally, analysis of fly activity has relied on single-beam (SB) infrared (IR)-based detection in *Drosophila* Activity Monitors (DAM), although ultrasonic waves, and more recently, various tracking-based approaches have been used to monitor continuous activity (Zimmerman et al., 2008; Faville et al., 2015; Donelson et al., 2012; Gilestro and Cirelli, 2009; Shaw et al., 2000). The traditional SB-IR systems measure fly movement by recording breaks of a single IR beam located near the center of a thin glass tube. Lack of any beam crossings over a 5-min period is interpreted to reflect a sleep-like state. The simplicity of this system, and its previous use in circadian biology have resulted in this becoming the most prevalent method to measure fly sleep (Pfeiffenberger et al., 2010a,b). One potential drawback of this approach is that it only detects movement at a single location in an arena, potentially overestimating sleep (Zimmerman et al., 2008; Faville et al., 2015; Gilestro, 2012). In addition, traditional SB DAM acquisition is unable to determine location preference within a recording chamber and cannot recognize small movements during periods of quiescence. To address these concerns a new generation of DAM monitors have been developed that detect movement throughout the entire arena using 17 distinct IR beams. Similar to the SB system, activity is recorded from flies housed in glass pyrex tubes; however, MB acquisition is advantageous in that it is able to more accurately resolve the fly's location within an arena. To date, differences in acquisition sensitivity and accuracy between these IR-based systems have not been investigated.

Video recording (Zimmerman et al., 2008; Gilestro, 2012) or tracking (Donelson et al., 2012) provide alternatives to infrared-based monitoring, and powerful freeware or commercially available software can be used to analyze rest-wake activity following video acquisition (Donelson et al., 2012; Gilestro and Cirelli, 2009). Video acquisition also provides increased accuracy, but throughput can be limited by data analysis and the lack of standardized commercially available recording systems (Gilestro, 2012). Similar to IR-based methods, previous studies involving tracking-based approaches have recorded fly behavior within the confines of a glass pyrex tube. While similarities in experimental methodology allow for direct comparisons between systems, conclusions thus far have been primarily limited to data generated within the standardized enclosure used for IR-based recordings.

Flies modulate sleep in accordance with numerous environmental and life-history traits, including age, mating status, feeding state, and arena size (Griffith, 2013; Bushey et al., 2011; Yurgel et al., 2014). For example, starved flies suppress sleep, and sleep becomes fragmented in aged animals (Keene et al., 2010; Koh et al., 2006; Metaxakis et al., 2014). These manipulations often affect sleep duration as well as sleep architecture, including the timing and length of individual sleep bouts (Zimmerman et al., 2008). While there is a growing appreciation for the importance of sleep modulation in response to environmental changes, the effectiveness of different sleep acquisition systems and the contributions of arena-shape, size, and activity detection method in measuring environmental and life-history dependent changes in sleep have not been systematically investigated.

In this study, we directly compare the ability of three distinct acquisition systems – a novel video tracking system, and SB- and multi-beam (MB)-IR systems – to measure sleep across a number different environmental and life-history conditions. To standardize video recording, we have designed an inexpensive recording chamber that can be used to track rest-wake activity using an open-arena and commercially available software or freeware. We test flies at different ages and varied feeding and mating states to investigate context-dependent differences between the two IR-based recordings systems and video tracking. These findings demonstrate certain advantages of the MB IR-based system and highlight versatility of video monitoring by examining sleep in different size arenas. We also describe a modified sleep preference assay where flies display a preference for daytime sleep in a dark environment. In agreement with previous findings, our results indicate the greatest differences between SB-IR DAM and video tracking when measuring sleep architecture (bout length and number); however, all systems are able to detect changes in sleep duration and architecture in the context of aging, starvation and mating status, indicating that in most experimental conditions the SB-IR system is sufficient for accurately measuring sleep duration. Therefore, these studies suggest video tracking and multi-beam systems provide increased resolution of sleep architecture across contexts, while all three systems are sufficient for measuring environment and life history-dependent changes in sleep.

## RESULTS

**Fig. 1.**
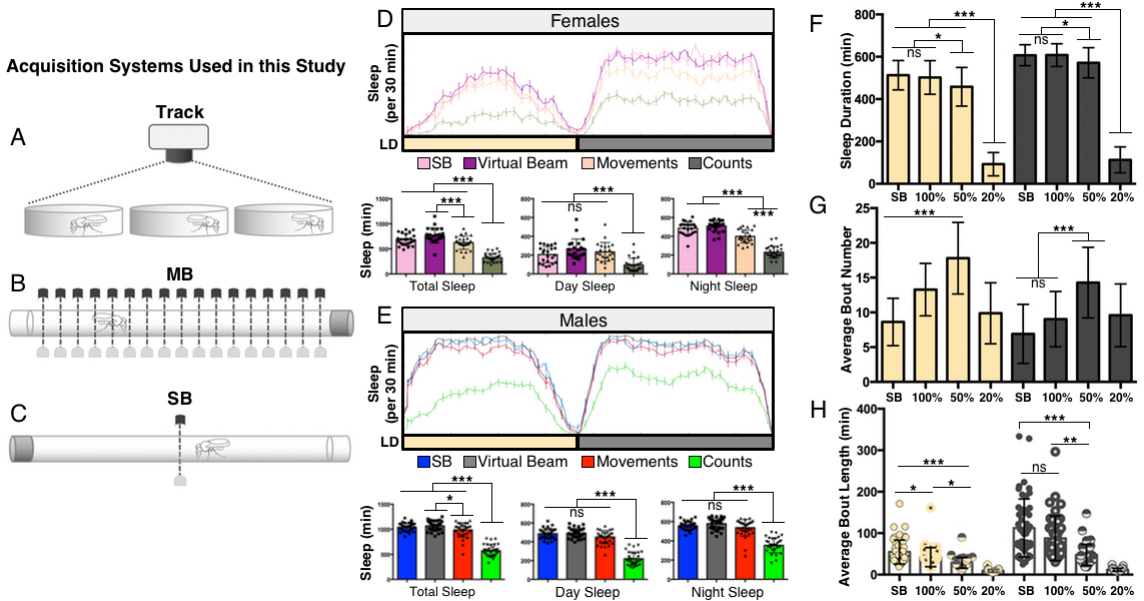
**Establishing activity parameters for video-tracking software.** (A-C) Schematic of the three acquisition systems used in the current study. Activity was recorded in *iso31* virgin male flies for 24 h on sucrose/agar using video tracking in 24 well tissue culture plates (Track), singlebeam IR (SB-IR), and multibeam IR (MB-IR). (D-E) In all panels, sleep was measured over 24 h using the multibeam (MB) DAM acquisition system, except for single beam analysis (SB). (D) Virgin female flies: upper panel – the MB-center-beam (purple) was specifically analyzed for sleep and compared to the SB-IR system (pink). Quantification of sleep over 24 h revealed no significant differences between the two readouts. Analyzing Movements (orange) and Counts (olive green) from all 17 IR detectors in the MB system revealed a significant reduction compared to single beam detection. Bottom panels – quantification of total, daytime, and nighttime sleep for each acquisition mode. (E) Male flies: upper panel – the MB-center-beam (grey) was specifically analyzed for sleep and compared to the SB-IR system (blue). Quantification of sleep over 24 h revealed no significant differences between the two readouts. Analyzing Movements (red) and Counts (green) from all 17 IR detectors in the MB system revealed a significant reduction compared to single beam detection. bottom panels – quantification of total, daytime, and nighttime sleep for each acquisition mode. Note, the Counts detection setting is significantly more sensitive than Movements for both sexes and appears unable to accurately detect bouts of quiescence compared to the traditional SB-IR system (see text for more details). (F-H) In all panels, sleep duration was calculated by analyzing 24 h video recordings of flies housed on sucrose/agar in 24-well tissue culture plates, except for single beam analysis (SB). (F) No differences were observed between 100% FBL and SB-IR. Daytime sleep duration was significantly lower when 50% FBL threshold analysis was compared to 100% FBL and SB-IR, while nighttime sleep comparing 100% and 50% FBL was at the threshold of significance. Using 20% FBL results in a significant decrease in sleep compared to both 50 and 100% FBL. (G) Using 50% FBL to detect movement resulted in higher day and night bout number compared to 100% FBL and SB-IR. Bout number was greater in daytime than nighttime when 100% FBL, 50% FBL, or SB-IR were used to determine activity. (H) Bout length was reduced during the day and nighttime when 50% FBL was used to determine activity. Overall bout length was reduced in 50% FBL threshold analysis compared to 100% FBL and SB-IR. Bar graphs are presented as means±s.d. *P<0.05; **P<0.01; ***P<0.001; ns, not significant. See Materials and Methods and Table S2 for details on statistical analysis.

Multiple systems have been used to measure sleep-wake activity in *Drosophila*, including video tracking, and SB- and MB-IR-based approaches (Fig. 1A-C); however, a direct comparison between all three systems across multiple variables and conditions is lacking. We first sought to define sleep parameters in the MB-IR and video tracking systems.

### Defining experimental parameters

#### MB parameters

Whereas the commonly used SB-IR acquisition software from TriKinetics (http://www.trikinetics.com) detects fly activity using beam-breaks from a single IR beam through the center of a glass tube, the TriKinetics MB system and Acquisition Software detect activity from 17 IR beams located along the length of the cuvette arena offering the ability to acquire more sophisticated activity data and parameters; these include Counts, Movements, and Dwell Time. In the ‘Movement’ detection setting, activity is determined when a fly moves from a given beam to an adjacent beam; in other words, intra-beam activity is not registered. On the other hand, ‘Counts’ are registered by each beam as the fly enters its path. If the fly crosses the path of a single beam, only a single count will be registered, while multiple counts may be recorded in succession if the fly remains within a beam (TriKinetics MB operating notes). Thus, the ‘Counts’ setting is presumably more sensitive, recording both intra- and inter-beam activity. Finally, extracting Count data only from the center beam within the arena is likely to produce data similar to the traditional SB system.

We first compared sleep acquired from the center beam of the MB-IR monitors (using MB Count settings), to that of the traditional SB-IR monitors. As predicted, sleep values from the center beam of MB-IR monitors did not differ from the SB method in both female and male flies (Fig. 1D,E; *P*>0.11, *P*>0.80). We next compared the MB Counts and Movements detection settings, utilizing all 17 beams of the MB, to that of a center beam. In both females and males, total sleep duration across 24-h using ‘Movements’ was less than that observed using the center beam of the MB system (Fig. 1D, *P*<0.001; Fig. 1E, *P*<0.05). Compared to SB-IR and Movement detection setting, lower amounts of sleep were observed using the ‘Counts’ detection, suggesting that Counts may be a more sensitive method for detecting activity (Fig. 1D,E; *P*<0.001). However, it is unclear if this intra-beam activity registered by ‘Count’ detection accurately reflects movement while the fly is awake or minor twitches and wing flicks while the fly remains asleep. Previous studies have determined that movement detection at even 20-33% of the fly's full body length over-represents activity (Faville et al., 2015; Donelson et al., 2012). Therefore we chose to use the ‘Movement’ detection setting when analyzing our MB sleep data.

#### Video/tracking parameters

In an effort to standardize *Drosophila* video tracking, we developed a custom-designed, cost-effective analysis chamber to allow for recordings from universally available fly arenas (Fig. S1, Table S1). This system provides high-resolution video of fly activity with autonomous temperature and light regulation.

We first sought to calibrate our video-analysis system to differentiate between true activity associated with wakefulness and smaller movements, or noise, associated with the tracking software. We compared sleep, bout number, and bout length using the 100%, 50%, and 20% FBL thresholds for sleep detection as previously described in a different tracking system (Donelson et al., 2012); we also compared these findings to data generated by the SB-IR system. Consistent with previously published data, the 20% FBL threshold drastically underestimated sleep due to numerous artifacts of activity detected as movement when computer generated tracks are compared with manual scoring of movements (Donelson et al., 2012) (Fig. 1F and data not shown). Also in agreement with previous findings, day and night sleep duration calculated using 100% FBL as criteria for movement did not differ from that obtained using a SB-IR system (*P*>0.90), but was significantly different from the 50% FBL threshold (Donelson et al., 2012) (Fig. 1F, Table S2; *P*≤0.05). Unlike the 100% FBL threshold, the 50% FBL threshold resulted in significantly enhanced day and night bout number compared to the SB DAM system (Fig. 1G; *P*<0.001). Artifacts of movement using the 50% detection thresholds were not detected when Ethovision-determined activity tracks were visually compared to fly movement determined by manually scoring individual flies (data not shown), indicating that this is an accurate reflection of activity. These data suggest that the 50% FBL threshold is more sensitive than the 100% FBL threshold in discriminating between bouts of activity and inactivity. There was also a significant reduction in night bout length observed using the 50% FBL threshold compared to the SB DAM, while this effect was not observed using the 100% FBL detection threshold (Fig. 1H; *P*<0.001). Because determining movement as 50% FBL appears to accurately measure activity, this threshold was used during this study.

### Comparing acquisition systems – standard conditions

**Fig. 2.**
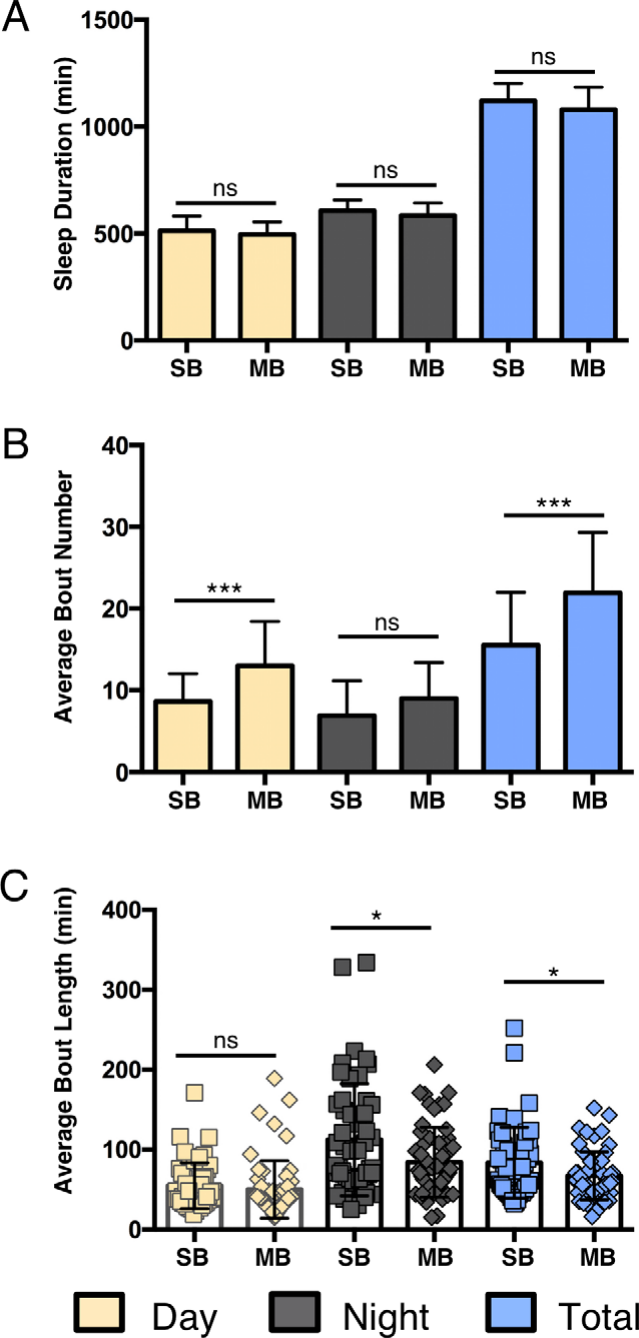
**Comparison of baseline sleep parameters between single beam and multibeam infrared-based acquisition systems under standard conditions.** For all panels, yellow bars represent daytime sleep, grey bars represent nighttime sleep, and blue bars represent total sleep over a 24-h period. (A) No differences in daytime sleep (yellow), nighttime sleep (grey) or total sleep duration (blue) were detected between SB and MB IR systems. (B) Daytime, nighttime, and total bout numbers were significantly increased in the MB DAM system compared to SB DAM. (C) Daytime bout length did not differ significantly between the MB and SB acquisition systems. Night and total bout length was significantly greater in the SB-IR than MB-IR acquisition. Bar graphs are presented as means±s.d. *P<0.05; **P<0.01; ***P<0.001; ns, not significant.

We next wanted to directly compare the SB and MB-IR acquisition systems by measuring sleep duration and architecture in male *iso^31^* isogenic flies. No significant differences were observed between the two IR systems for daytime (*P*>0.82), nighttime (*P*>0.58), or total (*P*>0.054) sleep duration (Fig. 2A, Table S2). To determine whether systems differentially detect sleep architecture, we examined bout number and bout length from data acquired in each system. The MB system detected significantly greater numbers of total sleep bouts than the SB-IR system, with a larger contribution from daytime differences (Fig. 2B, Day: *P*<0.001, Night: *P*<0.02; Table S2). These data suggest an increase in the number of IR-beams provides enhanced sensitivity in detecting disruptions in sleep. Daytime bout length did not differ between the systems (*P*>0.47), while nighttime bout length was higher in the SB-IR system (Fig. 2C; *P*<0.02). Likewise, analysis of total sleep revealed significantly greater bout length in SB-IR acquisition (*P*<0.03). Thus, under standard experimental conditions the MB-IR provides enhanced resolution over the SB-IR system in analysis of sleep architecture, while the systems do not differ in analysis of sleep duration. These data suggest that sleep bouts are likely more fragmented than what was originally observed from data collected with the traditional SB-IR system.

### Comparing acquisition systems – changes in environment and life-history

#### Mating status influences sleep parameters

**Fig. 3.**
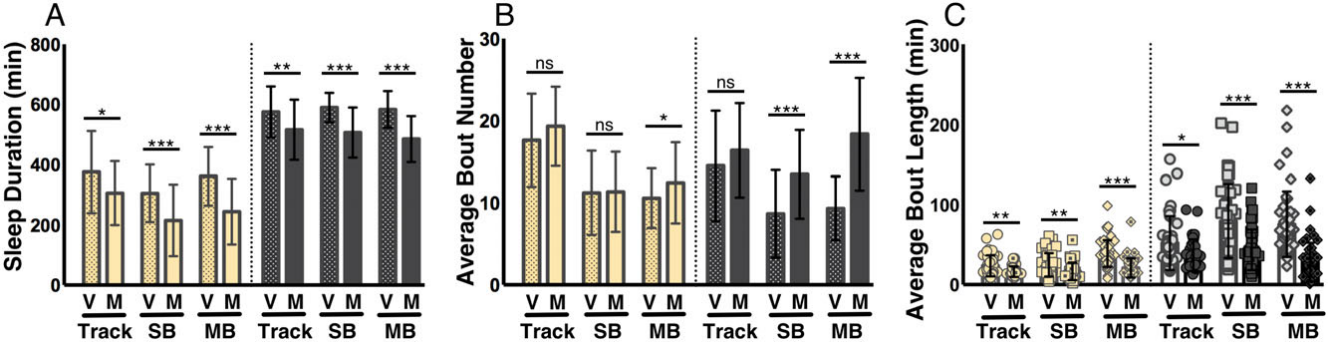
**Detecting effects of mating status on sleep.** For all panels, V=virgin and M=mated. Yellow bars represent daytime sleep and grey bars represent nighttime sleep. (A) All three acquisition systems detected reduced daytime and nighttime sleep in mated females compared to age-matched virgins. (B) No differences in daytime bout number were detected across acquisition systems. An increase in the number of nighttime sleep bouts was detected using both IR-based approaches, but not tracking. (C) Likewise, both IR systems, but not tracking, detected reduced average sleep bout length in mated flies. Bar graphs are presented as means±s.d. *P<0.05; **P<0.01; ***P<0.001; ns, not significant.

Female *Drosophila* modulate sleep in accordance with mating status (Zimmerman et al., 2012; Isaac et al., 2010). Previous results collected using the SB-IR DAM system demonstrated reduced daytime sleep in mated female flies compared to age-matched virgins (Zimmerman et al., 2012; Isaac et al., 2010). Mating status also affects many other behaviors including feeding and egg laying (Vargas et al., 2010; Ribeiro and Dickson, 2010), which may confound sleep detection in a SB system because animals might shift their location in the tube. We sought to determine whether mating-induced changes in sleep could also be detected across all three acquisition systems. Indeed, we observed reduced daytime sleep duration in mated females for the tracking system and both IR systems (Fig. 3A; *P*<0.02, *P*<0.001), suggesting that each analysis system is sufficiently sensitive to detect post-mating changes in daytime sleep. Nighttime sleep duration was also reduced across all three systems (Fig. 3A; *P*<0.01). Taken together, these findings indicate all three systems are generally sufficient to detect mating status-dependent changes in sleep duration.

Next, we investigated mating-induced changes in sleep architecture to determine whether each system is sufficiently sensitive to detect differences between virgin and mated female flies. Mating led to significant increases in bout number as detected by both IR acquisition systems (Fig. 3B; *P*<0.001), but this increase was not significantly different for video tracking (Fig. 3B; *P*>0.05). Note, while there was a significant difference between the duration of day sleep (previous section, Fig. 3A), no significant differences were detected in the number of daytime sleep bouts between virgin and mated flies using tracking or SB (Fig. 3B; *P*>0.16), though modest increases were seen in the MB system (*P*<0.05). Moreover, both day and night bout length were significantly reduced in mated flies using all three systems (Fig. 3C; *P*<0.017). Taken together, these data support the conclusion that mating affects the duration and architecture of sleep and indicate the IR-based recording systems are sufficient to detect these changes.

#### Comparing acquisition systems under starved conditions

**Fig. 4.**
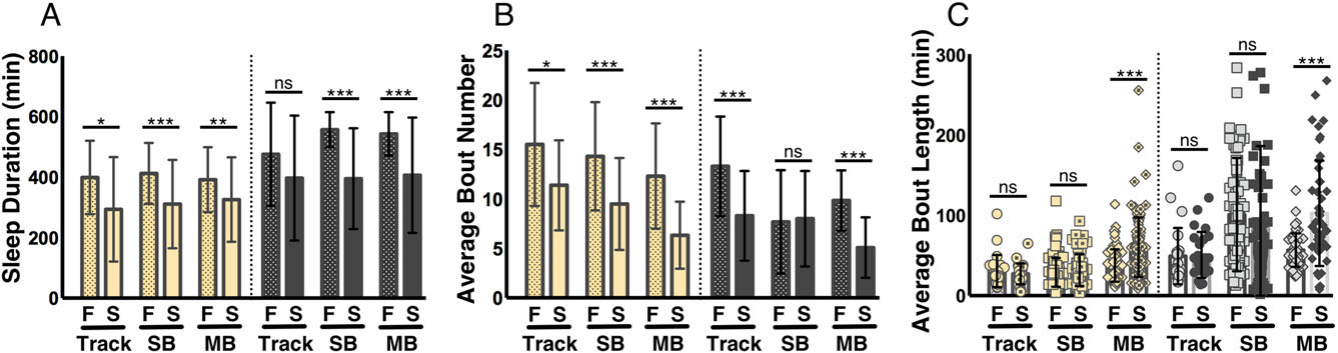
**The efficacy of acquisition systems in detecting food-deprivation induced changes in sleep.** For all panels, F=fed and S=starved. Yellow bars represent daytime sleep and grey bars represent nighttime sleep. (A) All three acquisition systems detected reduced daytime sleep under starved conditions while only the IR-based systems detected starvation-induced decreases at night. (B) A significant decrease in daytime sleep bout number was detected for all acquisition systems; only tracking and MB detected decreases in bout number at night. (C) Only MB analysis detected significant changes in average bout length during the daytime and nighttime. Bar graphs are presented as means±s.d. *P<0.05; **P<0.01; ***P<0.001; ns, not significant.

Starved flies potently suppress sleep and increase activity, presumably to forage for food (Keene et al., 2010; Thimgan et al., 2010; Mattaliano et al., 2007; Lee and Park, 2004) and we sought to determine the ability of each acquisition system to detect sleep changes in response to starvation. Virgin female flies were either starved on 1% agar or fed a diet of 5% sucrose and monitored for sleep over 24 h. To date, studies examining food deprivation in flies have compared fed and starved flies in a chamber where food is placed on one end of the chamber. The tracking system we describe differs because food or agar medium is placed along the entire surface of the chamber. Reduced sleep duration in flies housed only on agar was detected by all three acquisition systems during the day (Fig. 4A; *P*<0.017). Decreases in nighttime sleep were also detected by both IR-based systems (Fig. 4A; *P*<0.001) and trended towards significance using the tracking method (*P*>0.15). In agreement with previous findings examining starvation-induced sleep suppression with the SB-IR system, bout number was significantly reduced during the day and night using all three systems (McDonald and Keene, 2010) (Fig. 4B; *P*<0.02), except for the SB-IR system, which failed to detect a significant decrease at night (*P*>0.68; Fig. 4B). Interestingly, no significant differences in bout length were detected in the tracking (*P*>0.046) or SB-IR system (*P*>0.19), while day and night bout length is significantly increased in the MB-IR system (Fig. 4C; *P*<0.001), raising the intriguing possibility that arena size or shape, as well as resolution of movement detections, influences starvation-induced changes in sleep. In summary, all three systems detect starvation-induced changes in both sleep duration and aspects of sleep architecture.

#### System sensitivity in detecting age-dependent sleep changes

**Fig. 5.**
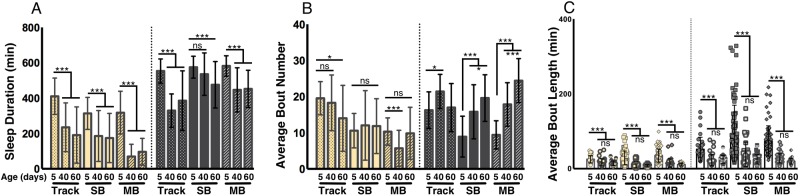
**Detection of aging-dependent changes in sleep.** For all panels, 5, 40, and 60=age of flies in days. Yellow bars represent daytime sleep and grey bars represent nighttime sleep. (A) Both day and nighttime sleep duration were reduced in flies aged 40 or 60 days compared to young 5 day old female flies across all three systems with the exception of nighttime sleep in 40 day old flies in the SB DAM system. (B) A significant increase in nighttime bout number in flies aged 40 or 60 days was detected across all three acquisition systems, with the exception of 60 day old flies in the tracking system. Average daytime bout number was not changed for any of the acquisitions systems. (C) Both daytime and nighttime bout length were reduced across all three acquisition systems in 60 day old flies compared to young 5 day old flies. Bar graphs are presented as means±s.d. *P<0.05; **P<0.01; ***P<0.001; ns, not significant.

Aged female flies display shortened and fragmented sleep (Zimmerman et al., 2008; Koh et al., 2006; Metaxakis et al., 2014). We tested age-dependent changes in male and female sleep using all three acquisition systems by examining sleep duration, bout number, and bout length of 5, 40, and 60 day old flies. Consistent with previous reports, 40 and 60 day old virgin female flies slept significantly less during the day than young 5 day old virgin female flies across all three acquisition systems (Fig. 5A; *P*<0.001) (Zimmerman et al., 2008; Koh et al., 2006; Metaxakis et al., 2014). All three systems also detected age-dependent decreases in nighttime sleep amounts between 5- and 40/60-day old virgin female flies (*P*<0.001), except for the SB-IR system, which only detected differences between 5- and 60-day old flies (*P*<0.01) (Fig. 5A; *P*>0.08 between 5 and 40). Therefore, in this situation, the SB-IR system appears to be less sensitive than the MB-IR and tracking systems in detecting age-dependent changes in sleep duration.

All three systems detected increased night bout number in 40 day old female flies compared to young 5 day old flies (Fig. 5B; *P*<0.02). Both IR-based systems also detected increased bout number in 60-day old flies; however, this is not significant using the tracking system (Fig. 5B; *P*>0.89). All three systems also revealed significantly shortened day and night bout length between young and old female flies, which confirmed the shortened sleep observed in female flies (Fig. 5C; *P*<0.001). Therefore, all three sleep systems are sufficient to detect age-dependent changes in sleep architecture in female flies.

The effects of aging on sleep are sexually dimorphic (Koh et al., 2006), so we also sought to examine the ability of each system to detect age-related sleep changes in male flies. No significant differences in day sleep were observed in 5, 40, and 60 day old male flies in any of the three systems (Fig. S2A; *P*>0.21), suggesting that aging does not affect daytime sleep in males. A significant reduction in nighttime sleep between 5 and 40 day old male flies was detected by all three acquisition systems (Fig. S2A; *P*<0.01); however, a significant reduction in nighttime sleep was only observed using the tracking system when comparing 5 and 60 day old flies (Fig. S2A; *P*<0.001). Day and night bout number increased in 40-day old male flies compared to 5 day old flies across all systems, suggesting each system is sufficiently sensitive to detect age-related changes in sleep bout number (Fig. S2B; *P*<0.01). The finding that, in many cases, sleep duration and architecture differ significantly at 40 days, but not 60 days, reveals dynamic modulation of sleep throughout the aging process. Significantly reduced day and night bout length were also detected using the IR systems in aged male flies (40 or 60 days old) compared to young flies (5 days old) (Fig. S2C; *P*<0.01). In summary, all three systems indicate that aging results in reduced sleep duration and fragmented sleep in females (Fig. 5A-C), while predominantly modifying sleep architecture in males (Fig. S2A-C).

#### Highlighting the versatility of video monitoring

**Fig. 6.**
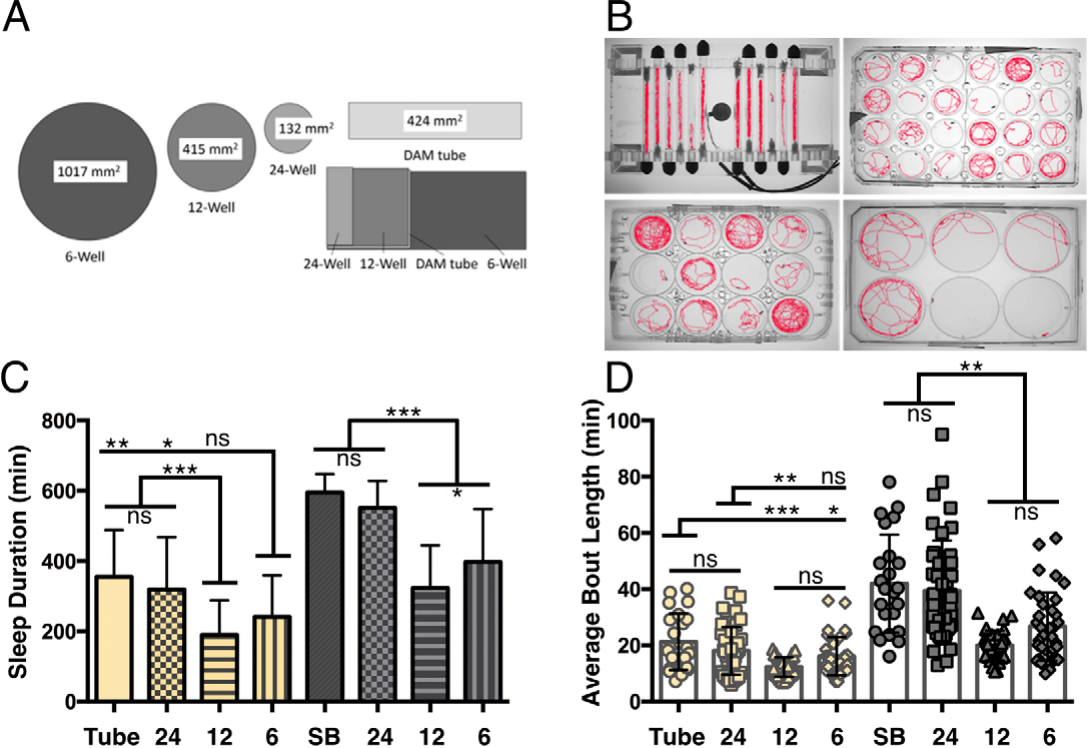
**Effects of arena size on sleep.** (A) Sleep was measured using video tracking in standard DAM tubes or 24, 12 and 6 well tissue culture plates with increasing area size. (B) Images of flies being tracked in DAM tubes or 24, 12, and 6 well tissue cultures plates. (C) Flies slept significantly less during both the day and nighttime when housed in 12 well or 6 well plates, than when housed in smaller 24 well plates or DAM tubes. (D) Average night-time bout length was reduced in 12 well and 6 well large arenas and daytime bout length is reduced in 12 well arenas compared to smaller DAMS tubes and 24 well plates. Bar graphs are presented as means±s.d. *P<0.05; **P<0.01; ***P<0.001; ns, not significant.

Video monitoring provides the flexibility to vary the size and shape of testing chambers. We investigated whether sleep is modulated in accordance with arena dimensions by measuring sleep within arenas of different sizes including 24, 12 or 6 well tissue-culture plates with an inner surface volume of 132 mm^2^ for 24 well plates, 415 mm^2^ in 12 well plates, and 1017 mm^2^ in 6 well plates (Fig. 6A). For comparison, we also included sleep measurements in DAM Tubes with an inner surface volume of 424 mm^2^. Flies slept significantly more in DAM tubes and 24-well plates during both the day and night compared to 12 and 6 well plates over the 24 h assay, suggesting that arena size is inversely related to sleep duration (Fig. 6C, *P*<0.04, *P*<0.001; Table S2). Interestingly, day and nighttime sleep duration did not differ between the DAM tubes and 24 well plates, despite a ∼3-fold difference in surface area, indicating a strong contribution of arena shape or food location to sleep duration (*P*>0.69; *P*>0.39). No differences in bout number were detected during the day or night for any of the four arenas shown (data not shown). The relationship between arena size and sleep appears to be primarily reflected in bout length because daytime bout length was significantly reduced in 12 well plates compared to both 24 well plates and DAM tubes (*P*<0.01), and nighttime bout length was reduced in both 12 and 6 well plates compared to smaller DAM tubes and 24 well plates (Fig. 6C; *P*<0.001). Taken together, these findings reveal that flies modulate sleep in accordance with arena size and shape highlight the ability of tracking systems to measure environment-dependent changes in sleep.

**Fig. 7.**
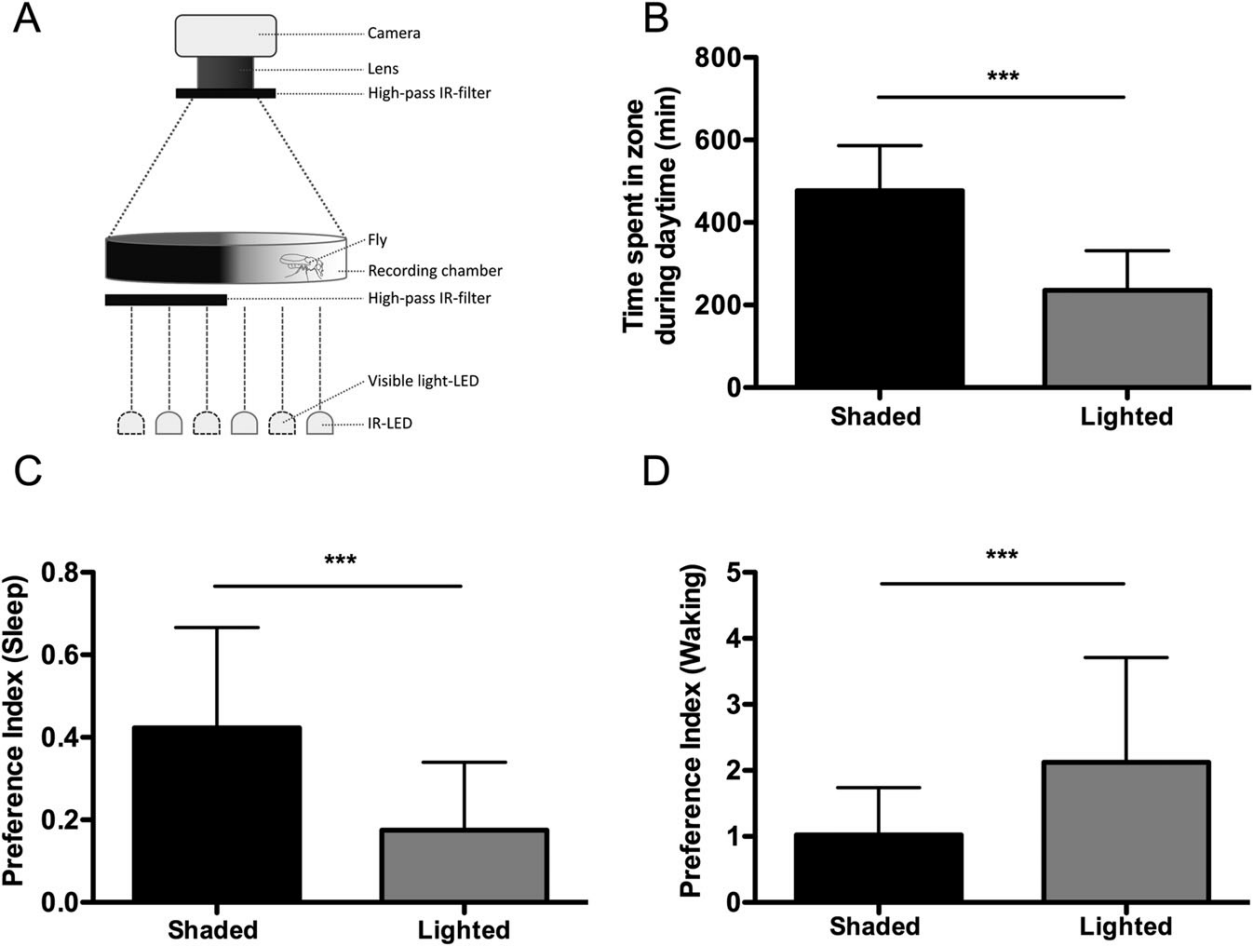
**Flies prefer to sleep in dark areas.** (A) Sleep was measured using video tracking of flies in circular sleep arenas where an IR-pass filter was used to block light in half of the arena. Total sleep and activity were determined for each side of the arena during the 12-h light phase. (B) Flies spent significantly more time in the dark areas than light. (C) Flies spent significantly more time sleeping in the dark area than the light area. (D) Flies spent significantly more waking time in the light half of the arena. Bar graphs are presented as means±s.d. ***P<0.001.

The flexibility of tracking systems can also be used to examine place-dependent sleep preference. Flies sleep less in constant light conditions compared to standard 12:12 light-dark cycles, and display a preference for sleeping in shaded or dark locations (Shang et al., 2008; Rieger et al., 2007). A previously developed assay measured sleep in tubes containing light, dim, and dark regions, with food located on one side of the tube (Rieger et al., 2007). To simplify this assay we generated a half lit arena by placing visible light blocking filters under 50% of a bottom-illuminated 6 well tissue-culture plate and measured sleep during the light phase (ZT0-ZT12) (Fig. 7A). Flies were housed on agar media containing 5% sucrose along the entire bottom of the plate, preventing food location from influencing preference. Flies spent significantly greater time sleeping in the dark portion of the plate (Fig. 7B; *P*<0.001) consistent with data from (Rieger et al., 2007), which suggests that flies prefer to rest in dimly lit or shaded locations. We should note that in contrast to the previous study, which simultaneously investigated an interaction with food location, flies in our study had access to the agar/sucrose nutrient source for the duration of the experiment. Importantly, when the time spent in each zone is subdivided into periods of sleep and wake, flies spent significantly more time sleeping in the dark region, while spending more time awake in the light region (Fig. 7C,D; *P*<0.0001). Therefore, flies display a preference for sleeping in shaded or darker locations, while preferring to spend waking time in more brightly lit locations. Together, the above experimental paradigms showcase the flexibility of video tracking-based acquisition of sleep and provide the opportunity to examine the biological basis for sleep place preference.

## DISCUSSION

Tracking systems and MB monitoring are capable of detecting movement throughout an arena and have the potential to be more sensitive than the traditional SB-IR DAM system in detecting sleep. The results from this study provide a detailed comparison of behavioral quiescence as measured across sleep acquisition systems. Our results indicate that under standard conditions measurements of sleep architecture obtained using MB (‘Movement’ parameter; Fig. 1D,E, Fig. 2) and our video tracking system (with a 50% FBL criterion, Fig. 1F-H) are more sensitive than SB-IR. These findings suggest that MB-IR and tracking systems may be advantageous for detecting qualitative changes in sleep, while these systems may only be marginally advantageous for examining total sleep duration. This feature may be important for mutations or environmental factors that specifically modulate sleep architecture. For example, mutations in the gene *amnesiac* do not impact total sleep duration, but result in fragmented sleep (Liu et al., 2008). Likewise, high-density larval rearing reduces bout number and increases bout length in wild-type, but not *amnesiac* mutant flies (Chi et al., 2014). Therefore, the tracking and MB-IR systems may be preferable to SB-IR for experiments specifically focused on examining how a genetic mutation or context impact more intricate dynamics of sleep. However, while we find the SB-IR system slightly overestimates sleep duration compared to video tracking (at 50% FBL, Fig. 1F), all three systems are generally able to detect differences in total sleep duration and architecture specifically associated with changes to mating status, nutrition, and age. Therefore, the SB-IR system is adequate for most sleep experiments and the simplicity of this system may make it preferable to MB-IR and tracking systems.

### Factors to consider in future experiments

#### Data load, analysis, and cost

Both the SB- and MB-IR monitors provide binary detection of fly activity. Therefore, these systems do not require the powerful computational processing and large data storage space required by tracking and video-recording systems, providing advantages in certain situations. With that said, the MB files also contain copious amounts of information and the .txt files can become quite large and cumbersome. Therefore, the MB-IR system provides an intermediate between tracking and SB-IR computer and processing power should still be taken into consideration. Additionally, due to the sheer amount of data collected, particularly in the case of high-throughput screens, analysis can be laborious. We have written customized Excel formulas and macros to make data analysis for the MB-IR system more efficient and manageable. However, in many situations, the advantages of increased resolution of behavioral tracking and sensitivity may be outweighed by potential complications in analysis. Finally, researchers should consider the cost of each system. Currently, the SB-IR monitors are significantly less expensive than the MB-IR monitors and can hold twice as many flies.

Although a number of other recording systems have been used for analysis of sleep behavior, in this study we describe a custom-built fly recording chamber that utilizes Ethovision XT 9.0 tracking software for analysis of fly activity. This commercially available program provides accessibility and a simple graphical user interface (GUI). Less expensive tracking versions have been developed as freeware that are open source and may provide increased flexibility and customization (Zimmerman et al., 2008; Donelson et al., 2012; Gilestro and Cirelli, 2009). While the tracking box described here is relatively inexpensive (Table S1), and one could use free software to acquire track recordings, the Ethovision Package is relatively expensive. Therefore, there are many options available depending on the research requirements and budget for projects involving tracking. However, we favor standardization across the sleep video-tracking field (see below).

Many tracking systems described to date for sleep analysis are limited by the inability to track multiple flies within an arena. This prevents researchers from examining multiple animals simultaneously to look at the effects of social interactions on sleep behavior. However, it should be noted that a number of programs, for example ctrax and JAABA, have been developed for the simultaneous tracking of multiple animals within a single arena (Branson et al., 2009; Swierczek et al., 2011; Kabra et al., 2013). While these systems provide increased flexibility, they are computationally demanding and are generally not used for long-term behavioral analysis or high-throughput screening.

#### Arena size and shape

Previous video-tracking experiments have relied on similar experimental arenas to that of the SB-IR; in other words flies remained confined to glass tubes and activity was detected using a ‘virtual beam’ in lieu of a single IR beam (Zimmerman et al., 2008; Faville et al., 2015; Gilestro, 2012). This provided the ability to directly compare data using tracking and IR-based acquisition from animals within the same experimental data set. These studies concluded that SB-IR overestimates sleep, and suggest that inclusion of additional beams would reduce detected periods of inactivity and more accurately measure sleep. The current study provides the added benefit of also comparing MB-IR to the other systems. Surprisingly, under standard conditions, no significant differences were detected specifically in daytime or nighttime sleep duration between the two IR systems (using Movement detection settings, Fig. 2), suggesting the MB-IR system does not increase the resolution of sleep duration under standard conditions. Alternatively, the Counts detection setting appears to increase sensitivity; however, it is unclear if all detected movement can be classified as wakeful activity. As mentioned previously, differences between SB-IR and MB-IR were detected for bout number and bout length (Fig. 2C), suggesting the MB-IR system may be particularly useful for sleep analysis examining differences in sleep architecture.

It is also worth noting that in contrast to previous video-tracking studies using flies housed in glass tubes and a virtual beam to detect activity, our study combines the advantage of tracking flies in an open arena. Importantly, similar to observations made from virtual- or IR- based DAM based approaches, we are able to detect context-dependent changes in sleep duration and architecture using this open-arena strategy. However, when specifically comparing nighttime bout number within the mating and aging experiments (Fig 3B, Fig 5B), increases observed in both single-beam or multi-beam systems are not apparent when tracking in plates. These findings raise the possibility that sleep architecture changes associated with life-history status in IR-based systems could be specific to the size of the arena, and highlight the importance of choosing arena size and acquisition method that are appropriate to the sleep variable under investigation.

In this study, we also find that mated flies sleep less in larger arenas. This in turn provides a model for examining the integration of spatial cues and exploratory behavior with sleep. Previously, when tracking activity was measured from a DAM tube, flies appeared to make fewer long-distance movements across the center of the tube, while the majority of their waking time was spent near the food source and sleep location was dispersed throughout the tube. We also observe similar place preferences using the MB-IR system (D.S.G., personal communications; data not shown). Consistent with this idea, it was recently reported that rest:wake activity can be dramatically different depending on where the virtual beam bisected the tube (Faville et al., 2015). Together, these data demonstrate that fly activity is not equally distributed all along the tube length. Therefore, it should be noted that, in certain situations, the accuracy of the SB-IR systems might be strongly influenced by tube placement in relation to the single infrared beam within the system. If the beam randomly ends up recording from a location closer to the food side of the tube, results could be skewed or misinterpreted. By using video tracking or MB-IR analysis, researchers would be able to avoid this potential pitfall. Furthermore, the increased resolution of the MB-IR and tracking systems could be used to specifically examine mechanisms underlying this place preference.

Finally, we extend previous studies examining location preference during sleep by providing flies an arena that is lit from below only on one side of the arena. We find that flies spend the majority of time sleeping on the dark side, supporting the idea that there is an active preference for sleeping in dimly-lit locations. While previous studies have used similar approaches to examine short-term place preferences (Reiger et al., 2007), we extend results to longer-term analysis. This assay in combination with previous work examining sleep location preference highlights the flexibility of tracking systems and opens up a new avenue for investigating spatial, in addition to temporal sleep dynamics. For example, sleep location reportedly differs in *CaskB* mutant flies raising the possibility that there are genetic modulators of sleep location (Faville et al., 2015). The systems described here may be applied for examining genetic modulators of sleep preference.

#### Parameters and thresholds

Traditionally, the *Drosophila* sleep field has relied on single-beam infrared detection to determine sleep measurements, a system originally used to investigate broad daily activity rhythms associated with circadian rhythms. New technologies, such as video recording and tracking, as well as MB-IR systems, are more commonly being used to advance our understanding and investigate precise characteristics of fly sleep that include place preference, posture, and arousal threshold (Zimmerman et al., 2008; Faville et al., 2015; Gilestro, 2012). As these new technologies are developed and utilized, standardizing parameters and methodologies that most accurately reflect a sleep-like state will be fundamentally important to unify conclusions and interpretations across research studies. Therefore, a challenge to all approaches will be to validate movement that reflects true wakefulness (see below).

Increasing the sensitivity of IR-acquired data acquisition associated with the MB system also brings the challenges of increased complexity. When originally comparing the ‘Counts’ to ‘Movement’ setting within the DAM MB acquisition software, we discovered that the Counts setting appears to overestimate fly activity and thus underestimate sleep. One interpretation is that the Counts detection setting is unable to detect true bouts of quiescence (similar to the 20% FBL threshold used in the video tracking; see below). On the other hand, Movement detection ensures that a fly is truly moving by detecting activity only when a fly moves from one IR-beam into an adjacent one. This rules out the possibility of artifacts associated with intra-beam detection of subtle movements while still in a sleep-like state. Therefore, we chose to use the Movement detection setting for MB analysis. Finally, it should be highlighted that the MB-IR system allows for the detection of place preference within a glass tube arena; something that cannot be accomplished with the SB-IR system. Therefore, the MB-IR system may provide greater resolution and flexibility of the SB-IR DAM, while avoiding the analysis and data storage difficulties of tracking systems.

Previous studies indicate that daytime sleep duration is significantly reduced in tracking systems, perhaps due to shorter bout length and small movements that are not detected in the SB DAM monitors (Zimmerman et al., 2008). In the Zimmerman et al. (2008) study, tracking data was recorded once every 5 s (or 0.2 frames per second). In another study, a system termed ‘Tracker’ recorded fly activity at 1 Hz (or one cycle per second) in a SB DAM system allowing for direct comparison of video analysis and infrared monitoring (Donelson et al., 2012). More recently, a tracking system termed DART (Faville et al., 2015) was used to track flies at 5 frames per second. For the purpose of our studies, we tracked flies at ∼30 frames per second. The results obtained here are largely in agreement with those reported in (Donelson et al., 2012), suggesting (1) lower tracking frame rate is sufficient in most situations for accurate activity detection, and (2) analysis performed at 50% FBL captures sleep differences compared to the SB-IR system. Similar to the previous study, we also find (1) little difference under standard conditions between SB-IR recordings and tracking analysis when 100% FBL is used as criteria, and (2) using a 20% FBL movement benchmark provides dramatically reduced sleep and bout number estimates while overestimating movement (this study and Donelson et al., 2012). Therefore, we used the 50% FBL threshold in this study. In general, we favor this concept of ‘percent of body length’ measurement as it will standardize analysis and allow for cross comparisons between studies. However, given the variability of tracking parameters stated at the beginning of this paragraph, it is clear that the *Drosophila* sleep community will only benefit from greater standardization of video tracking methodologies.

It is becoming obvious that fly sleep, like so many other behaviors, is extremely variable with differences in genetic background and environmental conditions exacerbating these inconsistencies. Additionally, parameters like sleep quality, quantity, arousal threshold, and latency can affect interpretations of baseline or mutant phenotypes. It was previously demonstrated that different ‘wild type’ backgrounds vary sleep duration drastically (Faville et al., 2015; Zimmerman et al., 2012). In this manuscript we perform all experiments in the isogenic *iso*^31^ background, and cannot rule out different results if experiments were performed in alternative commonly used backgrounds, such as Canton-S or *w^1118^*. From these findings it appears that all three acquisition methods are sufficiently accurate to study context-dependent changes in sleep. We caution that genetic variability and background will likely be just as much of a concern as method of data acquisition.

Taken together, we have directly examined sleep analysis in multiple recording systems across a number of environmental contexts. We find that SB-IR systems are acceptable for detecting aging, mating status, and starvation-dependent changes in sleep, but may slightly overestimate sleep consolidation compared to MB-IR or tracking systems. These data clearly establish that SB methodology can be used for qualitative approaches, such as performing high-throughput screens to identify potential sleep components. Once mutants are identified, tracking and MB approaches could be used to confirm and refine more intricate nuances of sleep analysis. These systems offer the advantages of determining place preference and changes due to environmental modifications. We also describe a custom-built recording chamber that allows for standardized and cost-effective video tracking and define multiple novel tracking assays that can be used to measure sleep using this system. Therefore, SB-IR systems provide an efficient and accurate method for measuring sleep duration, while video tracking allows for increased flexibility of behavioral paradigms and greater resolution of sleep architecture.

## MATERIALS AND METHODS

### Fly stock maintenance

All flies used in this study were the *iso*^31^ strain previously described (Ryder et al., 2004). The identical *iso*^31^ stock from the laboratory of A. Sehgal was used in all experiments to account for genetic variation and drift found in common laboratory strains (Faville et al., 2015; Zimmerman et al., 2012). Flies were raised and maintained on a standard cornmeal based diet and reared in 12:12 light-dark (LD) conditions at 40-60% humidity.

### InfraRed (IR)-based acquisition systems and behavioral analysis

All experiments using the DAM systems were performed at the University of Pennsylvania. *Drosophila* Activity Monitor-5 (DAM5), containing 5×65 mm glass pyrex tubes and MB5-MB *Drosophila* Activity Monitors (Trikinetics), containing 5 mm×80 mm glass pyrex tubes were used when performing SB and 17-beam recordings, respectively. In all cases except for aging experiments, 5-7 day old flies were entrained for at least two days to the appropriate light schedule, anesthetized using CO_2_ (except starvation experiments in which flies were anesthetized on ice), and loaded into the appropriate length DAM tubes. Sleep was recorded following a 20-24 h acclimation period. DAM tubes contained 5% sucrose in 1% agar, except for the starvation-induced sleep suppression experiments. Throughout each experiment monitors were housed in temperature and light controlled incubators (12 h light: 12 h dark, 25°, ∼40-60% humidity; Percival, I-36VL). Post-acquisition sleep analysis was performed using custom Excel macros to calculate sleep duration and architecture across 30-min time bins. Following the acclimation period, sleep analysis was completed using data across the first full LD cycle, starting at ZT0.

### Video-based acquisition systems and behavioral analysis

All video-based tracking experiments were performed at the University of Nevada Reno. Fly activity was recorded using an Ikegami ICD-49 camera (Ikegami Tsushinki Co.) housed in a custom-built observation chamber, which was placed in a temperature-controlled room. We developed a standardized and cost-effective recording chamber for video monitoring of fly behavior. The observation chambers consisted of an acrylic box with a camera housed on top (Fig. S1). All materials used to construct the recording chamber are described in (Table S1). The IR LEDs (Environmental Lights, part number irrf850-390-reel, San Diego, CA) were continuously illuminated, while the white LEDs (Environmental Lights Inc., part number dlrf3528-120-8-kit) were placed on a 12:12 light dark (LD) cycle using a manual timer (Intermatic, Model No. TN311). The observation chambers consisted of an acrylic box made of black acrylic (TAP Plastics). A diffusion plate was placed directly above and parallel to the top of the heat sink above the LED lighting. The bottom of the diffusion plate consisted of a 1.5 mm thick piece of white acrylic (TAP Plastics), which was placed 5 cm above the heat sink where the LEDs were located. Tissue culture plates and DAM tubes were centered on top of this diffusion plate in each observation chamber. An aquarium air pump was used to ventilate this portion of the observation chamber below the diffusion plate and above the aluminum heat sink. A computer fan was attached below the heat sink to ensure proper air ventilation below the observation chambers. The observation chambers were positioned 38 mm above a flat surface measured from the bottom of the heat sink. Unless otherwise noted, flies were recorded in standard 24-well plates filled with 5% sucrose in 1% agar to limit vertical movement. All video-based experiments were run at 25°C and 40-60% humidity.

Video was recorded using an IR-transmitting lens (Computar, Vari Focal H3Z4512 CS-IR 4.5-12.5 mm F 1.2 TV lens) at a resolution of 704×480 pixels at 29.97 frames per second using the Media Recorder 2.0 software (Noldus). An IR high-pass filter (Edmund Optics Worldwide, filter optcast IR part no. 46,620) was placed between the camera and the lens to block visible light from entering the camera lens to ensure evenly distributed lighting throughout the experiments. Video was captured using the Euresys PICOLO U4 H.264 board installed on a Dell Precision T3500 computer.

Video files were analyzed using Ethovision XT 9.0 video tracking software (Noldus). Sleep was calculated by measuring bouts of inactivity ≥5 min using a modified Excel macro (Duboué et al., 2011). Fly sleep and activity were analyzed in individual flies. Detection settings used the differencing method at level 43 in the detection settings option. Movement in the tracking system was defined as the fly moving ≥2.5 mm/s and ≥1.2 mm/s or approximately 100% and 50% of the Full Body Length (FBL) of a female fly in 1 s, respectively. Termination of movement was defined as the fly's activity dropping below the ≤1 mm/s and ≤0.5 mm/s for the 100% and 50% FBL thresholds, respectively. The 20% FBL threshold defined movement as the flying moving ≥0.5 mm/s and termination of movement as the fly moving ≤0.2 mm/s. The lowess smoothing filter was used at the default setting of 10 to reduce noise from tracks for all experiments. Similar thresholds were used to calculate sleep as previously described (Donelson et al., 2012). Sleep values were compared using 50% FBL unless otherwise noted (Fig. 1) (Donelson et al., 2012).

### Starvation-induced sleep suppression experiments

Virgin female flies aged 5-7 days were anesthetized on ice and loaded into 24 well plates or DAM monitors prior to ZT0. Half of the flies were placed onto 5% sucrose in 1% agar (fed group), while the other half of the flies were placed onto 1% agar alone (starved group). Sleep was then recorded for 24 h in both starved and fed conditions for all groups. Note, under the starved condition, most of the flies die between 24-48 h.

### Mating status sleep experiments

After being entrained to the appropriate light schedule, 5-7 day old virgin females were either paired with equal number of males overnight (∼16-20 h; mated) or group housed (virgin) before loading into the appropriate recording chamber the following day. All flies were provided a 24 h acclimation to recover from anesthesia. Sleep analysis was completed using data across the first full LD cycle, starting at ZT0.

### Aging experiments

Virgin female or male flies were isolated within 24 h of eclosion, separated by sex, and maintained under standard conditions. Flies aged to 40 or 60 days were flipped onto fresh vials every 2-3 days to prevent premature death from sticking to old food. At the appropriate age, flies were anesthetized and loaded into acquisition chambers the day before the start of data acquisition. Flies were provided a 24 h acclimation to recover from anesthesia. Sleep analysis was completed using data across the first full LD cycle, starting at ZT0.

### Arena size experiments

Mated female flies aged 5-7 days were used for these experiments because arena size-dependent changes in sleep were not observed in virgin female flies. Flies were loaded on ice into standard 24 well, 12 well or 6 well tissue culture plates (B&D Biosciences) or standard DAM tubes (Trikinetics, Inc.) prior to ZT0. The plates and tubes all contained 5% sucrose in 1% agar. The flies were acclimated for 24 h in the observation chambers before sleep was recorded. Fly activity was recorded for 24 h in the tracking boxes following acclimation.

### Light preference experiments

Virgin female flies aged 5-7 days were loaded on ice into 6-well tissue culture plates containing 5% sucrose in 1% agar and placed into the observation chambers prior to ZT0. Visible light blocking filters (Edmund Optics Worldwide, part 8″×10″ optical cast plastic IR longpass filter) were cut to size and placed along the bottom of the outer edge of each six well plate. The filters were positioned to block half of the visible light coming from below each well of a 6-well plate. Following a 24-h acclimation period activity was recorded in the tracking boxes for 24 h. Time spent in each half of the well was calculated for each fly by dividing the arena for each fly into equal size light and dark zones. A modified preference index was developed to account for sleep location and duration in individual zones. Sleep preference for each zone was calculated using the following equation for each individual fly: [(sleep in dark/light zone)/(total time spent in respective zone)]/[(total time in respective zone)/(total time in arena)], with time measurements in minutes. Waking preference was calculated using the same equation only using waking minutes instead of minutes sleeping for each individual fly. This equation prevents any bias based upon a fly's light-independent preference for one half of the arena.

### Statistics

Statistical analysis was performed using the GraphPad 6.0 (Prism) software package. Raw data and *P*-values for all figures are included in Table S2. For data shown in Fig. 1, a one-way ANOVA with analysis type (Fig. 1D,E): SB, Virtual Beam, Movements, or Counts; (Fig. 1F-H): SB, 100%, 50%, or 20% as a factor was run for each analysis period (Total, Day, or Night). Tukey multiple comparison post-hoc tests were performed based on the ANOVA results to determine which pairs were significantly different. For data shown in Fig. 2, an unpaired two-tailed Student's *t*-test with analysis type (SB versus MB) as a factor was run for each analysis period (Total, Day, and Night). For data shown in Figs 3 and 4, an unpaired two-tailed Student's *t*-test with analysis type (Fig. 3: virgin versus mated; Fig. 4: fed versus starved) as a factor was run individually for each acquisition system during each analysis period (Day, or Night). For data shown in Fig. 5 and Fig. S2, a one-way ANOVA with age (5/40/60) as a factor was run for each individual acquisition system during both analysis periods (Day or Night). Tukey multiple comparison post-hoc tests were performed based on the ANOVA results to determine which pairs were significantly different. For data shown in Fig. 6, a one-way ANOVA with analysis type (SB, 24-well, 12-well, and 6-well) as a factor was run for each analysis period (Day, or Night). Tukey multiple comparison post-hoc tests were performed based on the ANOVA results to determine which pairs were significantly different. For data shown in Fig. 7, an unpaired two-tailed Student's *t*-test was performed for each panel.
